# Historical Trends in Intraocular Lens Innovation Through an Analysis of United States Patents, 1972-2000

**DOI:** 10.7759/cureus.114028

**Published:** 2026-08-05

**Authors:** Evan Mason, Trevor Lin, Curtis E Margo

**Affiliations:** 1 Department of Ophthalmology, University of South Florida Morsani College of Medicine, Tampa, USA; 2 Department of Ophthalmology and Pathology and Cell Biology, University of South Florida Morsani College of Medicine, Tampa, USA

**Keywords:** food and drug administration, intraocular lenses, medical devices, medical technology, ophthalmic innovation, patents

## Abstract

One of the great advancements in ophthalmic surgery during the 20th century was the development of the artificial intraocular lens (IOL). There is remarkably little historical information on the trends in IOL innovation based on what inventors believed was worthy of patent protection in the United States (U.S.). We explored the topic of patentable IOL innovations by obtaining information through a global patent database. Patents issued for IOLs in the U.S. during the 20th century were identified.

A total of 583 IOL patents were issued in the U.S. last century. Based on creators’ justification of “unique” innovation, the single largest patentable category was lens design (264 (45.3%)), of which 197 (74.6%) were related to haptics. Time trends in some patent categories overlapped with the rise of phacoemulsification surgery. Just over half of the patents were owned by corporations (302 (51.8%)), while 254 (43.6%) were owned by individuals and 27 (4.6%) were owned by universities. Novel innovations in IOLs in the U.S. reached a peak during the last two decades of the 20th century.

Individuals owned a disproportionately high number of patents compared with other technology sectors. Only 156 patents (30.7%) were maintained throughout the full 20-year term. The authors speculate that the high rate of patent abandonment reflects a high rate of competing innovations.

## Introduction

Nearly 35 years passed between the first implanted intraocular lens (IOL) by Harold Ridley and the time when surgical implantation of IOLs became the standard of care in the United States (U.S.) [1,2]. Though the reasons for this delay have been debated, once IOLs became the preferred means of restoring refractive correction after cataract removal, the pace at which improvements in manufactured IOLs took place accelerated [3-5]. The first IOL was patented in the U.S. in 1958 (an anterior chamber lens). Then, from 1972 - when the next patent was issued - through 2000, 855 patents were granted for devices used in the replacement of the crystalline lens [6]. This number far exceeded the 316 patents for IOL surgery outside the U.S. during the same time period [6]. The nature and pace of these innovations remain largely speculative based on the IOLs that eventually reached the market.

Patents are issued by countries to protect creative innovation and have existed in Europe since the 15th century. Section 8, Article I of the U.S. Constitution gives Congress the authority to protect copyrights and patents and is a reflection of how important it was to the Founding Fathers to safeguard creativity [7]. A patent in the U.S. is issued for a device if it satisfies three criteria: (i) it must be substantially novel from what is publicly known; (ii) it must be demonstrably useful; and (iii) the novelty must be non-obvious to a qualified reviewer [7]. These criteria are not always easy to evaluate, as they require reviewers who have knowledge of a narrow field of historical technology, along with substantial insight into current cutting-edge technology.

In general, most technology patent applications deal with incremental advancements rather than revolutionary inventions. Because a minor variation in a pre-existing device might enhance its utility in incalculable ways, the task of a patent reviewer involves considerable judgment. A similar exacting duty presents itself when determining whether the novelty of a device is “non-obvious” to a qualified reviewer. There are relatively few qualified individuals who can reliably discriminate incremental yet truly novel changes in a narrowly defined field of technology. The same difficulty applies to finding people qualified to arbitrate trailblazing technologies who do not have conflicting interests. For technologies like IOLs, the need to find consultative expertise in fields outside anterior segment surgery (e.g., polymer science and optical engineering) is substantial.

The time from initial patent application to approval ranges from two to four years, which is long enough for some emerging technologies to become obsolete before patent approval [8]. IOLs also incorporate multiple technology domains, including structural design, biomaterials, and optical engineering. These practical and administrative impediments may explain why patent infringement litigation is not uncommon for commercially successful innovations [9,10]. Given these drawbacks, however, patents remain a reasonable surrogate for overall creativity in various spheres of technology. Within a defined time frame, the number of issued patents (and their options for maintenance) reflects the resourcefulness and inventiveness of an evolving area of technology.

The commercial success of a patentable device is another means of measuring the usefulness of an invention to society. Although this study could not directly assess commercial viability, novel inventions that enjoy market success usually have their patents maintained, making this metric a reasonable proxy for success. To gain a historical appreciation of the originality and inventiveness of the field of IOLs, we examined patentable innovations from 1972, when the second IOL patent was granted, through 2000, specifically examining patent types and temporal trends in innovation in the U.S.

## Materials and methods

Data source

Information on IOL patents was obtained from PATENTSCOPE, a global patent database maintained by the World Intellectual Property Organization (WIPO), an agency of the United Nations. The search focused on IOLs using the Cooperative Patent Classification (CPC) code A61F 2/16 from January 1, 1950, through December 31, 2000 [11]. A search was conducted using the internal query builder provided by the website, with the query string: DP: ((01.01.1950 TO 31.12.2000)) AND CPC:(A61F2/16) [12]. Results were filtered to include only inventions patented in the U.S. All patents found in this search were screened based on full-text descriptions, patent claims, and relevant drawings. Only patents directly and primarily related to IOLs were included. Patents related to manufacturing methods, implantation devices, and/or any other subject that did not primarily involve the implantable lens were excluded. Patents related to the surgical procedure rather than the lens were also excluded. Patents that lacked full-text descriptions were excluded.

Patent analysis

The results were downloaded into an Excel spreadsheet (Microsoft® Corp., Redmond, WA, USA) and categorized based on the primary new or novel patentable features of the IOL. Additional patent information was retrieved from the United States Patent and Trademark Office (USPTO), such as the publication date of the patent and the last “pay window” (how many times the patent holder paid to maintain the patent). Maintenance options occur at 4, 8, and 12 years; paying the 12-year fee preserves patent rights through 20 years. Patent maintenance payment options went into effect after December 12, 1980, with the passage of the Bayh-Dole Act, meaning that any patent submitted before this date was exempt from maintenance fees. Prior to 1980, the existing law stipulated a full term of 17 years without the possibility of extension [13]. The entity status of a patent refers to the size of the organization applying for the patent. Entities were classified as “undiscounted” (typically large organizations with >500 employees and not eligible for fee reductions) or “small” (eligible for reduced fees). Company or university ownership of patents was determined when they were listed as the current patent assignee.

Patent categorization

The names of individual applicants (individuals, companies, and universities) holding patents for IOLs were anonymized to reduce bias and emphasize patterns of innovation rather than those of specific entities. Lens innovations were reviewed by a single author and categorized based on what applicants argued was the primary patentable feature. These claims fell into eight general categories: (i) novel IOL biomaterial; (ii) novel lens design; (iii) novel means of optical correction; (iv) novel lens material additive; (v) novel method to prevent posterior capsule opacification; (vi) novel design to compensate for preexisting ocular pathology; (vii) integrated lens accessory; and (viii) phakic IOL (Table 1). Patents were subsequently placed into more highly defined subcategories based on the stated aims of the patent applications.

**Table 1:** Patentable or Novel IOL Categories IOL: intraocular lens; PCO: posterior capsule opacification

Category	Definition	Example
Novel IOL Biomaterial	Biocompatible material different from those existing, designed to improve safety or functionality	Elastic biomaterial that allows insertion through a small incision
Novel Lens Design	New and substantial alterations in the shape, configuration, or design of haptics, optics, or both that improve safety or effectiveness	Alteration of haptic shape or size that improves centration
Novel Means of Optical Correction	Novel methods of improving image quality of an artificial intraocular lens so that it improves existing methods, or more fully approaches the range of normal physiologic capabilities	Lens with more than one image focal point
Novel Lens Material Additive	Novel chemical additive combined with existing IOL biomaterial designed to improve safety or function of intraocular lens	Chemical additive to absorb ultraviolet light
Novel Method to Prevent PCO	Novel means of preventing lens epithelial cells from migrating along posterior lens capsule	Structural barrier design to block cell migration
Novel Design to Compensate for Pre-existing Ocular Pathology	Novel optical method or new lens design that improves visual function for persons with a preexisting ocular disease or disorder	Telescopic lens design for persons with macular pathology
Integrated Lens Accessory	A novel secondary device integrated into an existing lens to add utility or improve safety	Drug delivery component
Phakic Intraocular Lens	A novel lens designed to be used in eyes with a clear crystalline lens	Secondary lens implant with hinged haptics

## Results

**Figure 1:**
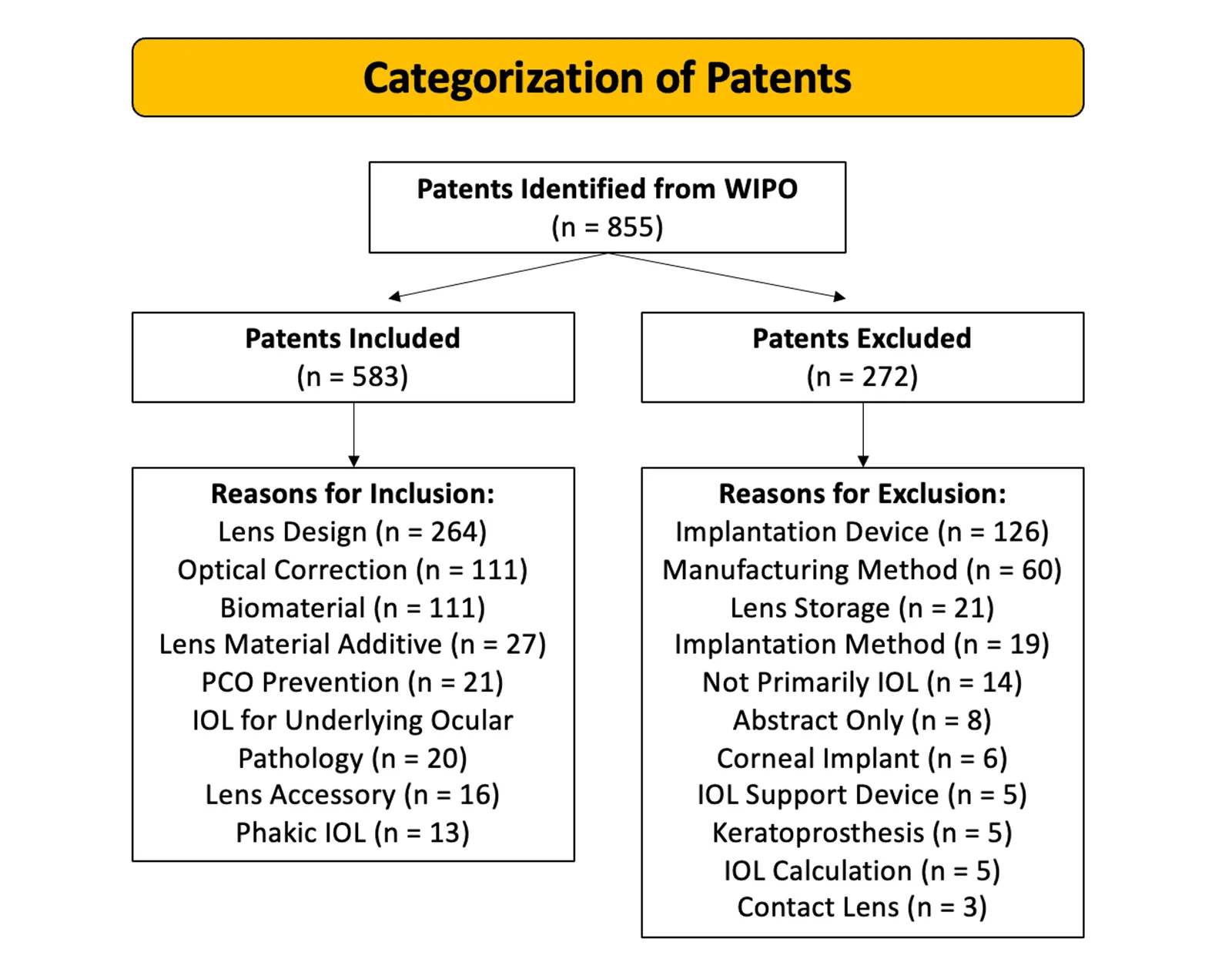
Flow Chart of IOL Patent Categorization IOL: intraocular lens; PCO: posterior capsule opacification; WIPO: World Intellectual Property Organization

The initial CPC-code search yielded 855 patents. Of these, 272 patents did not meet the inclusion criteria, leaving 583 patents for analysis. The first seven IOL patents (all before 1976) were excluded because they lacked sufficient descriptive detail and consisted only of a brief abstract. The four most common reasons for exclusion were as follows: (i) a novel device was used to insert the IOL (126 (46.3%)); (ii) novelty was limited to the method of manufacturing and did not alter the lens itself (60 (22.1%)); (iii) the patent was issued for lens storage material (21 (7.7%)); and (iv) the patent was granted for an instrument or surgical procedure related to implantation (19 (7.0%)) (Figure 1). 

The largest single category for the 583 IOL patents was novel lens design, totaling 264 (45.3%) (Table 2). The majority of these were for alterations in haptic design (197 (79.8%)). The next two largest categories were novel biomaterials and novel means of optical correction, each comprising 111 patents (19%). The major subset of novel biomaterials was fabricated for the optical portion (52 (46.8%)), of which 23 (44.2%) were primarily related to foldable properties. The largest subclassification of the novel means of optical correction was related to multifocal imaging (50 (45.0%)). Only one patent (2%) in this category was intended specifically for a small-incision wound. The remaining categories included lens material additives (27 (4.6%)), methods to prevent posterior capsule opacification (21 (3.6%)), novel designs to compensate for preexisting ocular pathology (20 (3.4%)), integrated lens accessories (16 (2.7%)), and phakic IOLs (13 (2.2%)) (Table 2). Of the 583 patents, only 77 (13.2%) either targeted or described innovation primarily for small-incision surgery (Table 2).

**Table 2:** Primary Patentable IOL Categories, 1976-2000* * Novel patentable innovations were placed in a single category and subcategory, but categories and subcategories were not mutually exclusive. Some could fall into two or more categories. ¶ Patent application includes or targets a lens intended for use with a small-incision surgical approach. # Designated by inventor to be additive to preexisting biomaterials. IOL: intraocular lens

Category	Total	Targeted for Small Incision Surgery¶
Novel IOL Biomaterial		
Adhesive	1	0
Drug additive	2	0
New haptic material	10	0
Properties for injection	9	0
Novel lens coating	13	0
New optic material	52	23
New haptic and optic material	13	4
Novel ultraviolet light blocker	11	0
Total: Novel biomaterial	111	27
Novel Lens Design		
New haptic design	197	16
New design related to inflatability	7	7
Novel IOL assembly	9	9
Novel optic design	51	15
Total: Novel lens design	264	47
Novel Means of Optical Correction		
Accommodating lens	20	0
Achromatic correction	2	0
Adjustable optical power	17	0
Aspheric correction	2	0
Extended depth of field	2	0
Holographic imaging	3	0
Multifocal lens designs	50	1
Novel “piggyback” coupling to primary lens	4	0
Incorporates stenopaeic optics	5	0
Toric correction	6	0
Total: Novel optical correction	111	1
Novel Lens Material Additive#		
Haptic additive	4	0
Color additive	8	0
Medication	2	0
Optic additive	4	0
Ultraviolet light blocker	9	0
Total: Novel lens material additive	27	0
Novel Method to Prevent Posterior Capsule Opacification		
Novel cytotoxic material	3	0
Related to electric current or field	1	0
Physical barrier	17	0
Total: Prevention of posterior capsular opacity	21	0
Novel Design to Compensate for Preexisting Ocular Pathology		
Glaucoma related	1	0
Novel design to use with spectacles	5	0
Prisms for image displacement	5	0
Telescopic optics for magnification	9	1
Total: Compensation preexisting ocular pathology	20	1
Integrated Lens Accessory		
Drug delivery apparatus	2	0
Intraocular pressure monitor	1	0
Lens hole plug	2	0
Novel lens marking	1	0
Novel device for mounting	10	0
Total: Lens accessory	16	0
Phakic IOL		
Novel haptic design	6	0
Novel optic design	5	1
Novel support mechanism	2	0
Total: Phakic IOL	13	1
Total	583	77

The annual number of IOL patents granted between 1976 and 2000 ranged from a low of 6 (in 1976) to a high of 47 (in 1987) (Table 3) and averaged 24.3/year. The annual total exceeded 30 in seven consecutive years from 1986 through 1992. The single largest category during these years was lens design, followed by novel means of optical correction (Table 3).

**Table 3:** Annual Number of IOL Patents by Innovation Category "-" indicates no patents issued in that category for the given year. IOL: intraocular lens; PCO: posterior capsule opacification

Year Issued	Biomaterial	Lens Design	Novel Optical Correction	Lens Material Additive	PCO Prevention	IOL for Underlying Ocular Pathology	Integrated Lens Accessory	Phakic IOL	Year Total
1976	1	5	-	-	-	-	-	-	6
1977	-	5	1	-	-	-	-	-	6
1978	3	5	-	-	-	1	-	-	9
1979	4	10	-	-	-	-	1	-	15
1980	1	5	-	1	-	-	-	-	7
1981	1	16	1	-	-	-	3	-	21
1982	1	12	-	-	-	-	1	-	14
1983	-	9	2	1	-	-	-	-	12
1984	5	17	1	1	-	-	-	-	24
1985	6	14	3	-	-	-	1	-	24
1986	5	31	1	-	2	1	1	1	42
1987	8	24	6	3	1	3	1	1	47
1988	7	15	7	3	2	1	1	1	37
1989	8	24	6	4	1	1	1	-	45
1990	10	11	11	-	2	1	-	-	35
1991	8	12	10	3	2	1	2	1	39
1992	6	9	17	2	-	1	1	1	37
1993	4	5	8	3	1	1	-	1	23
1994	4	3	4	1	1	2	-	1	16
1995	3	4	3	1	-	1	-	-	12
1996	5	6	7	-	-	-	2	1	21
1997	8	5	4	2	3	1	-	-	23
1998	6	7	7	1	-	2	-	1	24
1999	3	3	6	-	1	2	1	1	17
2000	4	7	6	1	5	1	-	3	27
Totals	111	264	111	27	21	20	16	13	583

Of the 583 patents, 508 (86.3%) were filed after December 12, 1980, when the maintenance fee “pay windows” were introduced (Table 4). Of these, 266 (52.4%) were maintained at four years, 206 (40.6%) at eight years, and 156 (30.7%) at 12 years. When maintained at 12 years, patent protection continued until the expiration date of 20 years. 

**Table 4:** Maintenance Rates of IOL Patents From Date of Issuance ¶ Total number represents patents after 1980, when the current maintenance system went into effect. § Payment at 12 years maintains the patent through the 20‑year term. * Overall patent device maintenance rates from 2012 [14]. IOL: intraocular lens

Category	Number of Patents Granted (%) ¶	Number Maintained at 4 Years (%)	Number Maintained at 8 Years (%)	Number Maintained at 12 Years (%) §
All IOL Patents	508 (100%)	266 (52.4%)	206 (40.6%)	156 (30.7%)
Novel Biomaterial	100 (100%)	60 (60.0%)	51 (51.0%)	40 (40.0%)
Novel Lens Design	210 (100%)	75 (35.7%)	51 (24.3%)	37 (17.6%)
Novel Means of Optical Correction	108 (100%)	73 (67.6%)	57 (52.8%)	44 (40.7%)
Novel Lens Material Additive	26 (100%)	17 (65.4%)	16 (61.5%)	12 (46.2%)
New Method to Prevent Posterior Capsule Opacification	21 (100%)	13 (61.9%)	9 (42.9%)	6 (28.6%)
Novel Design to Compensate for Preexisting Ocular Pathology	19 (100%)	12 (63.2%)	9 (47.4%)	8 (42.1%)
Integrated Lens Accessory	11 (100%)	7 (63.6%)	7 (63.6%)	5 (45.5%)
Phakic IOL	13 (100%)	10 (76.9%)	7 (53.8%)	5 (38.5%)
Number of Overall U.S. Technology Patents* (%)	542,825 (100%)	461,393 (85%)	325,689 (60%)	244,266 (45%)

All patents are issued to individuals, but ownership can be assigned to a company, corporation, or university once the patent is granted. Most IOL patents were owned by companies or corporations (302 (51.8%)) (Table 5). Only 27 (4.6%) were owned by universities. The remaining 254 patents (43.6%) were the property of individuals. Corporations exceeded individual ownership in six of the eight IOL innovation categories. The two categories in which individual ownership surpassed corporate ownership were lens design (145 versus 110, respectively) and prevention of posterior capsule opacification (13 versus 7, respectively) (Table 5). The disparity between individual and corporate ownership was greatest in the haptic design subcategory (112 vs. 78).

**Table 5:** Ownership of IOL Patents by Assignee Type IOL: intraocular lens; PCO: posterior capsule opacification; UV: ultraviolet

Patent Category	Corporate Ownership, Number	Individual Ownership, Number	University Ownership, Number	Total
Novel IOL Biomaterial	79	26	6	111
Adhesive	-	1	-	1
Drug Additive	2	-	-	2
Haptic	5	4	1	10
Injectable Lens	7	1	1	9
Lens Coating	9	4	-	13
Optic	37	11	4	52
Optic and Haptic	9	4	-	13
UV Blocking	10	1	-	11
Novel Lens Design	110	145	9	264
Haptic	78	112	7	197
Inflatable Lens	3	4	-	7
Intraocularly Assembled Lens	1	8	-	9
Optic	28	21	2	51
Novel Means of Optical Correction	58	47	6	111
Accommodating	6	11	3	20
Achromatic	-	2	-	2
Adjustable IOL	4	12	1	17
Aspheric	2	-	-	2
Extended Depth of Field	1	1	-	2
Holographic Optic	3	-	-	3
Multifocal	32	16	2	50
Piggyback IOL	2	2	-	4
Stenopaeic Holes	4	1	-	5
Toric	4	1	-	6
Novel Lens Material Additive	18	7	2	27
Haptic	2	2	-	4
IOL Coloring	7	1	-	8
Medication Release	1	1	-	2
Optic	3	-	1	4
UV Blocking	5	3	1	9
Novel Method to Prevent PCO	7	13	1	21
Cytotoxic	1	1	-	3
Electric Current	-	1	-	1
Physical Barrier	6	11	-	17
Novel Design to Compensate for Underlying Ocular Pathology	16	3	1	20
Glaucoma Related	-	1	-	1
IOL With Spectacles	4	1	-	5
Prismatic Design	4	-	1	5
Telescopic Lens Design	8	1	-	9
Integrated Lens Accessory	5	9	2	16
Drug Delivery	1	-	1	2
IOP Monitor	-	1	-	1
Lens Hole Plug	-	2	-	2
Lens Marking	1	-	-	1
Lens Mount	3	6	1	10
Phakic IOL	9	4	-	13
Haptic	5	1	-	6
Optic	3	2	-	5
Support System	1	1	-	2
Total	302	254	27	583

## Discussion

The federal government grants exclusive rights for the use and sale of new and useful products through patent laws. These laws vary by country but have several core features in common, such as time limitations. Patents are good markers for technological creativity and commercial success. Legally, they are intangible properties, and ownership can be sold or leased. As technology has advanced, the patent process has become increasingly nuanced, particularly in terms of satisfying the criteria of novelty, usefulness, and non-obviousness. Patent examiners determine “novelty,” for example, largely based on the persuasiveness of evidence presented in the application. A considerable burden of proof rests with the examiner to reject the claim of novelty [15]. Authorities have noted that this may create an applicant bias and can impede the ability to identify and reject unwarranted patents [15].

In the U.S., patents can be maintained for 20 years. They can also be abandoned (or not maintained) at 4, 8, or 12 years. Although there may be specific scenarios in which a patent owner may strategically allow a patent to lapse prematurely, it is generally in the owner's best interest to maintain the patent as long as its expected economic value exceeds the cost of maintenance. Of the 508 IOL patents granted in this study after December 12, 1980, 266 (52.4%) were abandoned at four years. Only 156 (30.7%) patents were maintained for 20 years (Table 4). Overall, these proportions are low compared with technology patents overall. A 20-year retrospective review of U.S. technology patents in 2012 found that 85% were maintained at four years, 60% at eight years, and 45% at 12 years (Table 4, bottom row) [14]. The reasons for the higher abandonment rate of IOL patents are speculative. One explanation may be the rapid evolution of cataract surgery and concurrent advances in material and optical engineering technologies, which can render lens innovations obsolete relatively quickly. In addition, marginal or incremental innovations may not be sufficient to gain traction in an increasingly competitive market. A third possibility is that safety or effectiveness concerns emerged after market introduction. Rates of post-marketing IOL recall have varied over time, with some lenses removed from the market before FDA intervention [16]. The reasons for post-marketing removal are often not publicly known [1,3]. However, FDA IOL recalls have been relatively few compared with implantable devices in other surgical specialties [17]. In all likelihood, some combination of these factors explains the high rate of IOL patent abandonment.

The annual number of IOL patents peaked between 1986 and 1992, with an annual average of 35.8 patents compared with the overall annual mean of 24.3. The majority of patents during this seven-year interval were in the categories of lens design (126 (50.4%)) and novel means of optical correction (58 (23.2%)). Perhaps the single most important surgical trend during this period was the rising popularity of phacoemulsification cataract extraction [18]. It appears that the surge in IOL patents roughly overlaps with this surgical transition period. One of the early drawbacks of phacoemulsification surgery was the need to enlarge the extraction wound to a size that allows lens implantation. The first foldable IOL entered the U.S. market in 1985-1986 [19-21]. Although premarket testing for biocompatibility was completed for this lens by Mazzocco in 1984, it was not FDA-approved for insertion when folded [22]. Only later, after further testing, could it then be folded and inserted through a small wound. This general advancement, however, served as a creative and commercial stimulus to develop other lenses that could fit through a small phacoemulsification wound. In addition to foldable lenses, some inventors developed designs that could be assembled or inflated in the eye through a small incision. Clearly, innovative trends in surgical technique breed innovations in IOLs.

The early 1990s witnessed an increase in novel methods of optic correction, including toric lenses and early multifocal lenses. The quest for an improved or ideal biomaterial beyond polymethylmethacrylate had been relatively consistent beginning in the mid-1980s. When surgical procedures demanded IOLs to egress small incisions, there was a surge in novel biomaterials that were optically transparent, had refractive capabilities, could be deformed, and had elastic properties. Beginning in the late 1980s, interest developed in lenses that could compensate for underlying ocular pathology, such as age-related macular degeneration. Other patentable innovations that appeared at this time and continued until the end of the century were methods to retard or prevent posterior capsular lens opacification and phakic IOLs (Table 3). 

Patent ownership data should be interpreted cautiously. Licensing, assignments, and subsequent commercial use are usually conducted privately and are not public records after patents are issued. The transactions of intangible private property, such as patents, can be conducted in private. The lack of transparency has proven frustrating for those interested in the follow-up of federally funded research under the Bayh-Dole Act, particularly when patents have resulted in high-priced medical products [23,24].

This study provides both a historical overview of what might be considered the most resourceful and productive period in the evolution of IOLs and a method of tracking domains of technological development in the future. There are limitations to the method of categorization used in the study, most importantly that the assignment of novel innovation rests with the inventor’s opinion and may not necessarily fit into a single major or minor category. For example, a new biomaterial can cause less light scatter and provide greater flexibility and elasticity. These features could potentially improve optical imagery and mechanical insertion through a small incision and thus fall into two categories. This categorization was performed by a single author, which could introduce another source of bias. The study also could not address why certain IOL patents had lapsed before their full term of protection. Another limitation is the inclusion of inventions patented solely in the U.S., thereby potentially excluding important innovations from other countries. This study was limited to IOLs patented in the U.S. for several reasons. First, the U.S. issues the largest number of IOL patents [6]. Second, laws governing patent regulations vary by country, making it difficult or even impossible to compare things such as maintenance rates. And third, critical information on these patents had to be retrieved through a national public domain database (USPTO), which in other countries is not always freely accessible. A final limitation of this study is that patented IOLs could not be correlated with their commercial success.

## Conclusions

In summary, the range of IOL innovations in the 20th century was broad, with the majority being issued for novel lens design, novel biomaterials, and novel means of optical correction. A substantial proportion of patents were owned by individuals and relatively few by universities. This percentage of medical device patents maintained by individuals, however, was unusual in the latter part of the 20th century, when manufacturers were generally better positioned to bear the cost and complexity of initiating and bringing novel surgical devices to market. The high abandonment rate of IOL patents compared with other technological domains likely reflects the premature obsolescence of many innovations and the rapid pace of newer (i.e., replacement) innovations. This study provides a more complete appreciation of the inventive contributions in IOL technology that occurred during the latter half of the 20th century.
